# Two cases of large cell neuroendocrine carcinoma of the gallbladder: case reports and systemic literature review

**DOI:** 10.3389/fmed.2026.1885339

**Published:** 2026-09-28

**Authors:** Yan Yang, Chunmei Zhang, Qi Liu, Wenyan Lin, Zhengjin Li, Lixian Zhao, Yun Pan

**Affiliations:** 1 Clinical Medical College, Dali University, Dali, Yunnan, China; 2 Department of Pathology, The First Affiliated Hospital of Dali University, Dali, Yunnan, China

**Keywords:** gallbladder, gallbladder neoplasm, large cell neuroendocrine carcinoma, neuroendocrine carcinoma, pancreatobiliary maljunction

## Abstract

As gallbladder large cell neuroendocrine carcinoma (GB-LCNEC) is extremely rare and highly aggressive, with limited reports in both domestic and international literature, we herein report two cases of GB-LCNEC accompanied by contiguous high-grade intraepithelial neoplasia of the overlying mucosa. One of the patients had pancreaticobiliary maljunction. Microscopically, the tumor cells were large and arranged in organoid, nested, palisading or trabecular patterns. They exhibited abundant cytoplasm, vesicular nuclei with coarse granular chromatin, prominent nucleoli, and mitotic figures exceeding 20 per 2 mm^2^. Immunohistochemically, both tumors showed diffuse positivity for the neuroendocrine markers chromogranin A (CgA) and synaptophysin (Syn), and both exhibited a Ki-67 proliferative index exceeding 20%. They demonstrated wild-type and aberrant (mutant) p53 expression patterns, respectively. Although the 2 cases reported in this study were confirmed to have space-occupying lesions in the gallbladder via CT/MRI, preoperative diagnosis was not achieved due to insufficient understanding of the disease. Through a systematic literature review, this study delineates the clinicopathologic and molecular features, diagnostic criteria, differential diagnoses, treatment, and prognosis of GB-LCNEC, as well as its potential pathogenesis/origin that may involve a multistep carcinogenic process of chronic inflammation–metaplasia–dysplasia–carcinogenesis. It aims to enhance clinical understanding of this rare disease and lay the foundation for future related research.

## Introduction

In the 2019 World Health Organization (WHO) classification and grading criteria for digestive system tumors, the hepatobiliary system was included in the overall framework of gastroenteropancreatic neuroendocrine neoplasms (GEP-NENs) for the first time (1, 2). In this context, the WHO released a dedicated classification for neuroendocrine neoplasms in 2022 (3). Neuroendocrine neoplasms (NENs) are a class of tumors originating from stem cells, characterized by the expression of neuroendocrine markers and the capability to produce bioactive amines and/or polypeptide hormones (4).

NENs can involve multiple organs throughout the body, most commonly the digestive tract (approximately 66%) and the lungs (approximately 31%) (5). In contrast, neuroendocrine neoplasms of the gallbladder (GB-NENs) are exceedingly rare, accounting for only 0.5% of all NENs and comprising about 2.1% of all gallbladder malignancies (6). Among them, well-differentiated ones are termed gallbladder neuroendocrine tumors (GB-NETs), which account for approximately 2%–3% of primary gallbladder neoplasms; poorly differentiated ones are called gallbladder neuroendocrine carcinomas (GB-NECs), accounting for about 4% of malignant gallbladder neoplasms (7). Based on histopathology, GB-NECs are further subdivided into small cell NEC (SCNEC) and large cell NEC (LCNEC). The U.S. Surveillance, Epidemiology, and End Results (SEER) database indicates that the incidence rate of GB-SCNEC is less than 0.74 per 100,000 annually (8). In contrast, GB-LCNEC is even rarer, with an incidence rate below 0.20 per 100,000 annually (9).

The first global case of GB-LCNEC was reported in 2000 by Papotti et al. (10). Although subsequent case reports of GB-LCNEC have been published (10–48), a unified understanding and standardized diagnostic and therapeutic protocol have not yet been established in the academic community. The reasons for this are as follows: (1) Early-stage disease lacks specific symptoms; (2) The imaging manifestations are non-characteristic and difficult to distinguish from other malignant gallbladder tumors; (3) Pathological differential diagnosis is prone to misdiagnosis as pure poorly differentiated carcinoma due to insufficient tissue sampling, incomplete immunohistochemical staining, or overlooking the diverse histological components within the tumor; and (4) There is currently no standard treatment regimen (9). Here we report two rare cases of GB-LCNEC and provide a brief literature review to enhance understanding of its clinical features.

## Case presentation

### Case 1

A 61-year-old female presented with abdominal pain and nausea. Abdominal ultrasound and CT performed at another hospital both indicated a gallbladder space-occupying lesion. Upon admission to our hospital, physical examination revealed no jaundice, palpable abdominal mass, or enlarged superficial lymph nodes. The patient denied anorexia and unintended weight loss, and preoperative liver function tests remained within normal limits. Her carcinoembryonic antigen (CEA) level was found to be elevated to 38.6 ng/mL, and repeated imaging confirmed a solid space-occupying lesion in the gallbladder (Supplementary Figure S1). She subsequently underwent laparoscopic radical cholecystectomy with R0 resection. The patient had an uncomplicated postoperative stay and healed well prior to discharge. Histopathological examination confirmed the diagnosis of (Gallbladder Body) Large Cell Neuroendocrine Carcinoma (LCNEC), with focal high-grade intraepithelial neoplasia of the gallbladder mucosa. Microscopic histopathological and immunohistochemical details are presented in Table 1 and Figure 1. The tumor invaded the perimuscular connective tissue of the gallbladder but did not penetrate the serosal layer. There was no evidence of liver infiltration, lymph node metastasis, or distant metastasis. The surgical margins of liver segments IVb and V as well as the gallbladder stump were all pathologically negative, and no regional lymph node metastasis was detected. Therefore, according to the 8th Edition UICC/AJCC TNM staging system for gallbladder cancer, the tumor was staged as pT2N0M0 (Stage II). The patient declined adjuvant therapy postoperatively and underwent routine surveillance with symptomatic management. Patients are required to return for follow-up every 3 months after surgery with tumor marker testing and imaging scans. No evidence of tumor recurrence or metastasis was noted during the first 7 months. At the eighth-month follow-up visit, the serum CEA level rose to 22.49 ng/mL, and imaging showed newly developed perihilar soft tissue density around the hepatic hilum, which was highly suspected to be local recurrence. Limited accessibility and high costs precluded somatostatin-receptor-targeted imaging; therefore, this patient did not receive DOTA-based DOTATATE-PET/CT throughout follow-up surveillance. Following thorough discussion, the patient and their family decided to transfer to a higher-level hospital for chemotherapy. Due to her advanced age, the patient could not remember the specific chemotherapeutic drugs. We only knew she received regular chemotherapy, and detailed treatment records were inaccessible via telephone follow-up. The total follow-up duration has reached 19 months since diagnosis, and the patient is currently in good general condition.

**Table 1:** Microscopic details of H & E and IHC of two cases.

Feature	Case 1	Case 2
H & E	The tumor exhibited an organoid or nested growth pattern, composed of large cells with abundant cytoplasm and nuclei that were round to polygonal with prominent nucleoli. Mitotic figures were frequent (>20/2 mm^2^), with no evidence of vascular, perineural, or lymph node invasion. High-grade intraepithelial neoplasia was identified in the local gallbladder mucosa. Both the hepatic resection margin and gallbladder stump were tumor-free.	The tumor cells were arranged in nested or patterns and composed of large cells with abundant cytoplasm, vesicular nuclei, and prominent nucleoli. Mitotic figures were frequent (>20/2 mm^2^). The tumor infiltrated the adventitial layer of the gallbladder wall, with no evidence of vascular, perineural, or lymph node invasion. The nodule at the gallbladder neck showed high-grade intraepithelial neoplasia of the gallbladder mucosa. The stump of the common bile duct was tumor-free.
IHC	Positive: CgA, Syn, CD56, CK7, CK19, CKp, CK8/18, CEA.	Positive: CgA (strong), Syn (strong), CK19 (focal), CK7 (focal).
	Negative: Vimentin, Hepatocyte, GPC-3.	Negative: CD56.
	Ki-67: 90%.	Ki-67: 70%.
	p53: Wild-type pattern.	p53: Aberrant strong and diffuse staining (mutant pattern).

**Figure 1:**
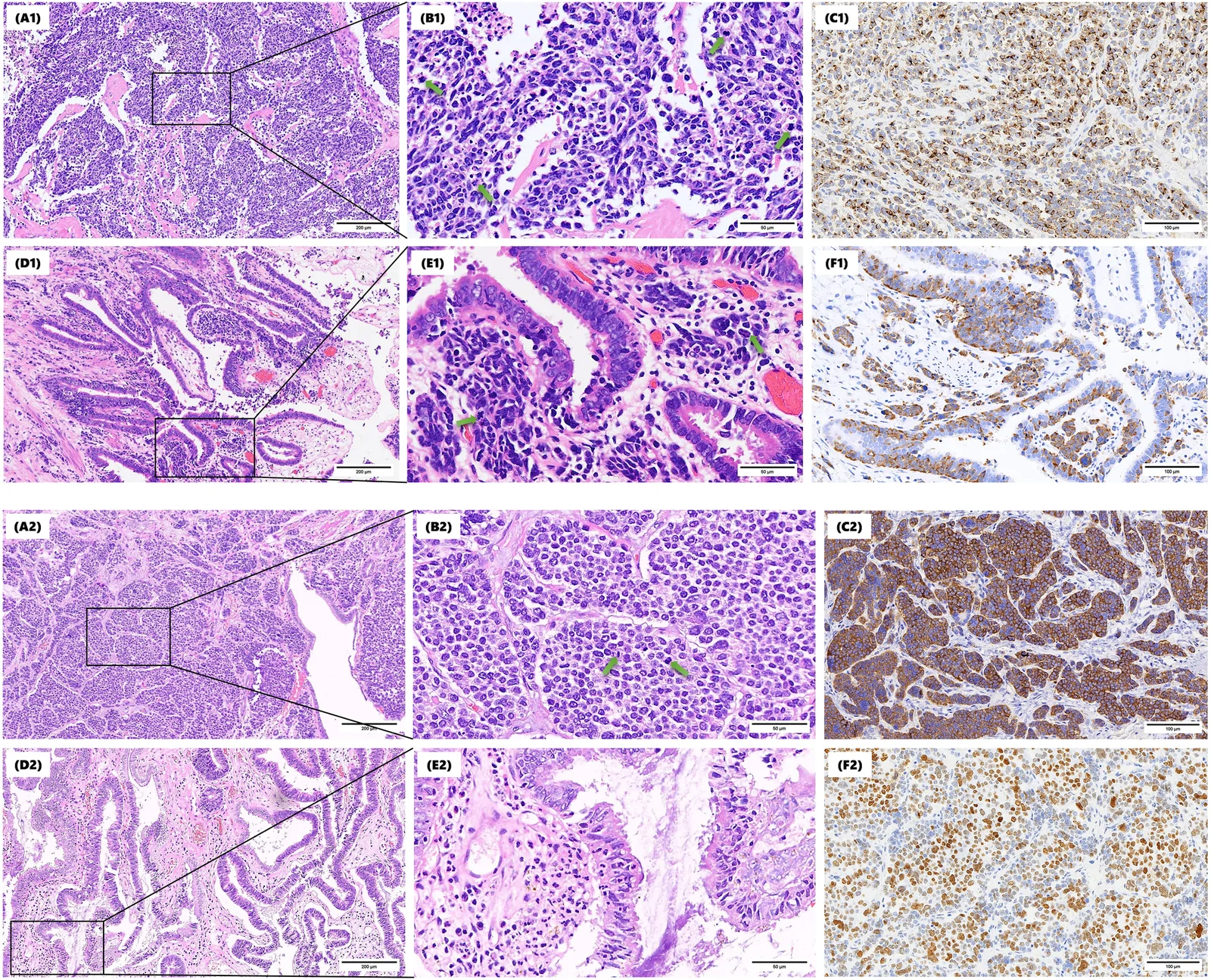
Histopathological (H & E) and immunohistochemical (IHC) findings of the two cases. Panels **(A1–F1)** correspond to Case 1: **(A1,B1)** The tumor shows a nested growth pattern, composed of large cells with abundant cytoplasm and nuclei that were round to polygonal with prominent nucleoli (H & E, ×100 and ×400, respectively); **(C1)** IHC showing CgA positivity in tumor cells (×200); **(D1,E1)** The overlying epithelium is replaced by low- and high-grade dysplasia with clear continuity between dysplastic glands and invasive carcinoma (H & E, ×100 and ×400, respectively); **(F1)** Scattered Syn-positive cells are observed in dysplastic glands and stroma (×200). Panels **(A2–F2)** correspond to Case 2: **(A2,B2)** The tumor shows an organoid growth pattern (H & E, ×100 and ×400, respectively); **(C2)** IHC showing Syn positivity in tumor cells (×200); **(D2,E2)** The mucosa surrounding the tumor shows intraepithelial neoplasia with chronic cholecystitis (H & E, ×100 and ×400, respectively); **(F2)** IHC demonstrates a mutant-type p53 expression pattern (×200). The green arrows point to atypical mitotic figures. IHC was performed using the EnVision method.

### Case 2

A 62-year-old female was admitted to the hospital due to abdominal pain. Upon admission, physical examination showed no jaundice, palpable abdominal mass, or enlarged superficial lymph nodes. She denied anorexia and unintended weight loss. Preoperative liver function tests and tumor markers (CA19-9, CEA and CA125) were all within normal limits. Imaging examinations revealed an enlarged gallbladder with a fundal space-occupying lesion (Supplementary Figure S1), along with pancreaticobiliary maljunction (PBM) and a choledochal cyst. Subsequently, she underwent laparoscopic excision of the choledochal cyst, cholecystectomy, and Roux-en-Y hepaticojejunostomy. The patient achieved smooth recovery with no surgery-associated complications. Histopathology indicated: (Gallbladder fundus) large cell neuroendocrine carcinoma, with high-grade glandular intraepithelial neoplasia identified in the surrounding gallbladder mucosa. Microscopic histopathological and immunohistochemical details are presented in Table 1 and Figure 1. Microscopically, the tumor cells infiltrated the adventitial layer of the gallbladder wall, and no evidence of distant metastasis was detected by imaging examinations. Liver segments IVb and V were not resected during the operation. Although all harvested regional lymph nodes were negative for metastatic carcinoma, surgical resection margin status could not be pathologically evaluated. Therefore, according to the 8th edition of the UICC/AJCC TNM staging system for gallbladder cancer, the tumor was staged as pT3N0M0 (Stage III). In retrospect, the surgical scope of this case failed to meet the guideline-recommended radical standard, which was classified as suboptimal surgical treatment. To reduce the risks of postoperative tumor recurrence and distant metastasis, we held consultations with the patient and her family. However, the elderly patient and her family refused extended radical resection, fearing that she could not withstand the physiological burden of extensive surgery. For this reason, the patient received five scheduled cycles of adjuvant etoposide–cisplatin chemotherapy postoperatively. Meanwhile, patients are required to return for follow-up every 3 months after surgery with tumor marker testing and imaging scans. At the most recent follow-up exceeding 15 months, no recurrence or metastasis has been detected.

We performed a literature search on PubMed, China National Knowledge Infrastructure (CNKI), and Wanfang Data from January 1, 2000, to December 31, 2025, using the following search terms: Gallbladder, Large cell neuroendocrine carcinoma, Neuroendocrine carcinoma, Gallbladder neoplasm, and Pancreatobiliary maljunction. Only English and Chinese literature was included. A total of 39 case reports were found. After reviewing all 39 case reports, other neuroendocrine neoplasms of the gallbladder were excluded. Finally, 43 cases of gallbladder large cell neuroendocrine carcinoma were confirmed in the present study (Table 2).

**Table 2:** Patient characteristics of 45 cases of large cell neruoendocrine tumor of gallbladder.

Case No.	Author[ref.]	Age/Sex	Presentation	Tumor location and size	Other component	Gall-stone	Cholecystitis	Thickened gallbladder wall	Tumor marker	Liver invasion	Metastasis	Regional lymphatic metastasis	Treatments	Prognosis, follow-up(month)
1	Papotti et al. (10)	65/M	ND	Fundus, 5 cm	None	+	ND	ND	ND	ND	+, Liver	None	cho + L + che	DPD, 14
2	Papotti et al. (10)	50/M	ND	ND, <1 cm	AC	+	ND	+	ND	ND	None	None	cho	DFS, 12
3	Jun et al. (11)	67/F	AP, AM	ND, ND	None	ND	ND	+	ND	+, Liver S4	+, Liver S6	none	che	DPD, 10
4	Jun et al. (11)	55/M	AP, JND	ND, ND	None	+	ND	+	ND	ND	+, Multiple lymph node	+	che	DPD, 1
5	Noske and Pahl (12)	81/F	AP, WL, JND, FNPF	Neck, 4.5 cm	AS	+	ND	+	ND	+	+, Femoral head	ND	PR	ND
6	Noske and Pahl (12)	55/F	BP, Fever, AP, JND	Fundus-body junction, 4.9 cm	AC + SCNEC	None	ND	ND	CA19–9 rose to 199 U/mL	None	None	None	Cho + PR	DFS, 20
7	Iype et al. (14)	58/F	AP	ND, 2 cm	None	+	ND	ND	ND	None	+, Liver portal lymph node	+	Cho + extended hepatectomy + Che	DFS, 16
8	Iype et al. (14)	85/M	AN, WL	Fundus, 1.5 cm	AC	+	ND	+	ND	None	+, Liver, peritoneum	ND	Cho + Che + PR	DPD, 21
9	Shimono et al. (15)	64/F	AP, AM	ND, 11.5 cm	None	ND	ND	ND	CA19–9 rose to 118 U/mL	+	+, Brain	None	MT: Che + Rad + L+γ Knife	DFS, 69
10	Yoon et al. (16)	38/F	WL, JND, AM	Fundus, 7.8 cm	ND, Biopsy-only	ND	ND	ND	CA19–9 rose to 79.9 U/mL; bile CA19–9 rose to 18,100 U/mL	+, Liver S4	+, Liver portal lymph node, bile duct junction	+	PR	DPD, 5
11	Lin et al. (17)	65/F	FG, AN, CSM	Body, ND	None	ND	ND	+	ND	+	+, Liver	ND	Radical Cho + L + HT + PR	DPD, 2
12	Sato et al. (18)	68/F	IF	Fundus, 3.5 cm	AC	+	ND	+	ND	+, Liver S4/5	None	+	Cho + L	DFS, 12
13	Mondolfi et al. (19)	48/F	AP	Fundus, 3.5 cm	AC	ND	ND	+	CA19–9 ↑ (the value ND)	+	+，Liver	+	Radical Cho + extended hepatectomy	DFS, >12
14	Al-Brahim and Albannai (20)	45/M	AP, AN, JND	Fundus, 2 cm	AC	+	ND	+	ND	+, Liver S5	+, Liver	+	Cho + Biopsy	ND
15	Russo et al. (21)	59/M	AP	Body, 4 cm	AC	+	+	+	ND	+	ND	+	Cho + L + Rad	ND
16	Samad et al. (22)	67/F	AP, JND	ND, 4.7 cm	None	ND	ND	ND	CA19–9↑ (the value ND)	+	+, Multiple	+	PR + Che	DPD, <2
17	Nakagawa et al. (23)	56/M	LEP (orbital metastasis at first diagnosis)	ND, 9 cm	None	+	ND	+	ND	+	+, Multiple	+	OT + Che + Rad	DPD, 36
18	Okuyama et al. (24)	64/M	AD, SLAP	Fundus, 2.5 cm	None	ND	ND	+	Normal	+	+, Multiple	+	Che	DPD, 22
19	Meguro et al. (25)	54/F	AP, JND	Fundus, body, >3 cm	AC	ND	ND	+	CA19–9 rose to 97 U/mL	none	+, ND	+	Radical Cho + PR	DFS, 24
20	Kamboj et al. (26)	50/F	ND	ND, ND	None	ND	ND	ND	CA19–9 rose to 407 U/mL	+	+, Multiple	ND	Che	DPD, 3
21	Kamboj et al. (26)	61/M	ND	ND, ND	None	ND	ND	ND	ND	+	+, Multiple	ND	No Che	DPD, 2
22	Liu et al. (27)	63/F	AP, Fever	Body, 2 cm	AC	+	+	+	Normal	None	None	None	Open Radical Cho + L	DFS, 12
23	Buscemi et al. (28)	76/F	AP	Fundus, 1.8 cm	None	+	ND	+	ND	+	+, Liver	+	Open Cho + Che + SSA	NPD, 5
24	Miyamoto et al. (29)	86/F	IF	Body, 2.5 cm	None	ND	ND	+	ND	None	None	None	Radical Cho	DFS, 6
25	Fujii et al. (30)	60+/M	AP	Fundus, 2.9 cm	None	ND	ND	+	Normal	+, Liver S4/5	None	None	Radical Cho + extended hepatectomy	DFS, 67
26	Millien et al. (31)	35/F	AP	Fundus, 10.8 cm	SRCC	None	ND	+	CA19–9 rose to 40.77 U/mL; CA125 rose to 33.8 U/mL	+	None	ND	Che + Rad	ND, ≥6
27	Moris et al. (32)	29/M	AP, N	Fundus, ND	None	+	+	ND	ND	None	None	None	Radical Cho + extended hepatectomy	DFS, 4
28	Peters et al. (33)	64/M	IF, HLF	Body, 2.5 cm	None	None	+	+	ND	+	+, Liver, lung	+	Cho + Biopsy	LTFU，≥2
29	Soin et al. (34)	62/F	AP, N, D	Fundus, 5.3 cm	AC	None	None	+	CEA rose to 6.8 ng/mL	None	None	None	Radical Cho + L + Che + Rad + Anti-HCV Tx	DPD, 2
30	Abutaka et al. (35)	67/F	AP	Body, ND	None	+	+	+	ND	+	+, Liver, lung	None	Cho + extended hepatectomy + PR + Che	DPD, 26
31	Shapera and Bitting (37)	65/F	AP	Fundus, body,2.5 cm	None	+	+	+	ND	None	None	None	Radical Cho + Che	DFS, 19
32	Chi et al. (36)	59/M	AP	ND, ND	None	None	None	+	ND	None	None	None	Radical Cho + Anti-tumor TCM	DFS，24
33	Xu et al. (38)	36/F	AP	Fundus, 4.3 cm	AC	+	+	+	ND	None	+, Liver, peritoneum	+	Cho + Che	DPD, 5
34	Tidjane et al. (40)	68/F	IF	Fundus, body, 3 cm	None	ND	ND	+	Normal	+, Liver S4b/5	+, ND	+	Radical Cho + extended hepatectomy + Che	DFS, 26
35	Ma et al. (39)	72/M	AP, WL	Fundus, 3.5 cm	AC + SC	ND	ND	+	CA19–9 rose to 210 kU/L	None	None	None	Radical Cho	DFS, 2
36	Shackelford et al. (42)	62/M	V, AP	ND, 3 cm	None	+	ND	+	ND	None	+, ND	+	Cho	LTFU
37	Du et al. (41)	ND/ND	ND	Neck, 0.7 cm	None	ND	ND	+	Normal	ND	+, Lung	None	Radical Cho + Che	ND, >9
38	Dhruv et al. (43)	72/M	C, AP	ND, 7.2 cm	None	+	ND	+	ND	+	+, Multiple	+	PR	ND
39	Fick et al. (44)	65/F	AP, AD, N, D	Fundus, 3.2 cm	AC	None	None	+	CA19–9 rose to 51 kU/L	None	None	None	Radical Cho + extended hepatectomy	DFS, ≥36
40	Liao et al. (45)	66/M	AP, WL, NV	ND, 1.6 cm	None	ND	ND	+	Normal	None	None	None	Cho + extended hepatectomy + Che	DFS, 14
41	Shaikh et al. (46)	73/F	AP, NV, Fever	Fundus, 2.0 cm	None	+	+	+	ND	None	None	+	Cho + Che	DFS, 36
42	Sah and Shrestha (47)	65/F	AP, WL, AN	ND, 7.0 cm	None	+	None	+	Normal	+	None	+	Open extended Cho	DFS, 6
43	Yuan and Chen (48)	45/F	AP	Body 3.1 cm; neck 1.5 cm	None	+	ND	+	ND	+	+, Hepatic hilar region	ND	Cho + Biopsy + PR	ND
44	Current study	61/F	AP, AD	Body, 4 cm	HGIN-Gb	+	+	+	CEA rose to 38.6 ng/mL	None	None	None	Radical Cho + L + Che	DFS, 7
45	Current study	62/F	AP	Fundus, 2 cm	HGIN-Gb	None	+	+	Normal	None	None	None	Cho + PR + Che	DFS, 15

AC, adenocarcinoma; AD, abdominal distension; AM, abdominal mass; AN, anorexia; AP, abdominal pain; AS, adenosquamous carcinoma; anti-tumor TCM, anti-tumor traditional Chinese medicine; Anti-HCV Tx, anti-HCV therapy; BP, back pain; biopsy, percutaneous liver biopsy; C, constipation; Che, chemotherapy; Cho, cholecystectomy; CSM, Cushing's syndrome manifestations; D, diarrhea; DFS, disease-free survival; DPD, death due to progressive disease; F, female; FG, fatigue; FNPF, femoral neck pathological fracture; HT, hormonal therapy; HGIN-Gb, high-grade intraepithelial neoplasia of the gallbladder; HLF, hepatic liver function abnormal; IF, incidental finding; JND, jaundice; L, limited hepatic wedge resection; LEP, left-eye proptosis; LTFU, lost to follow-up; M, male; N, nausea; ND, not described; NPD, non-progression-related death; OT, orbital tumor resection; PR, palliative resection; Rad, radiotherapy; Radical Cho, radical cholecystectomy; SSA, somatostatin analog; SRCC, signet-ring cell cholangiocarcinoma; SC, sarcomatoid component; SLAP, superficial lymphadenopathy; V, vomiting; WL, weight loss.

## Discussion

Based on the aforementioned overall classification framework for neuroendocrine neoplasms (NENs), neuroendocrine neoplasms in specific sites such as the gallbladder also follow the same classification logic. Histologically, GB-LCNEC resembles LCNEC arising in the lung and gastroenteropancreatic (GEP) system. GB-LCNEC typically demonstrates an aggressive growth pattern, characterized by diffuse involvement of the gallbladder and full-thickness wall invasion, frequently accompanied by lymphovascular and perineural invasion. Due to these features, it is crucial to distinguish it microscopically from other morphologically overlapping entities, including small cell neuroendocrine carcinoma (SCNEC), mixed neuroendocrine-non-neuroendocrine neoplasm (MiNEN), and poorly cohesive adenocarcinoma. This distinction relies on both morphological and immunophenotypic features (38), the diagnostic criteria of GB-LCNEC are shown in Supplementary Table S1 (1–3). Both cases in our study were diagnosed following the latest WHO guidelines. Consistent with the criteria listed in Supplementary Table S1, the tumors displayed characteristic organoid, nested, palisading, and trabecular architectures composed of large tumor cells with prominent nucleoli. Mitotic activity exceeded 20 mitoses per 2 mm^2^, and the Ki-67 labeling index was markedly elevated (90% and 70% for the two cases, respectively). Diffuse expression of neuroendocrine markers confirmed neuroendocrine differentiation. We excluded metastatic tumors based on imaging findings and immunohistochemical results. With adequate histologic sampling, SCNEC and MiNEN were also ruled out, leading to the definitive diagnosis of GB-LCNEC.

LCNEC, a high-grade neuroendocrine neoplasm, typically arises primarily in the lung and gastrointestinal system. However, it can also occur in other rare anatomical sites, including the female reproductive system (uterus, cervix), urinary system (urinary bladder), and biliary system (gallbladder). Among these, clinical reports of GB-LCNEC are particularly rare: since its first documentation by Papotti et al. in 2000 (10), only 43 cases have been recorded in domestic and international literature (10–48) (Table 2). Among 43 cases from the literature, some cases did not fully satisfy the criteria for pure GB-LCNEC according to the 5th edition of the WHO Endocrine and Neuroendocrine Oncology Blue Book, released in 2022 (3). To comprehensively reflect the historical reporting spectrum of the disease, illustrate the evolution of diagnostic criteria, and ensure data integrity and traceability, all cases—including those not fully meeting current criteria—have been summarized in Table 2 with corresponding annotations to facilitate reader consultation and distinction.

The pathogenesis of GB-NENs remains unclear and continues to be a focus of academic debate. Two main hypotheses exist: one suggests an origin from undifferentiated pluripotent stem cells (49), while the other, more strongly supported by clinical data (e.g., 47% of patients have gallstones, and 22% have a history of chronic cholecystitis, Table 2), proposes that chronic inflammation induces metaplasia, leading to neuroendocrine differentiation (33, 37). Notably, the patient in Case 2 presented with Type IA congenital biliary dilatation, a form of the anomalous union of pancreaticobiliary ducts (AUPBD), also known as pancreaticobiliary maljunction (PBM). According to previous literature (44, 50), PBM-associated biliary malignancy typically follows a multistep oncogenic pathway characterized by persistent chemical irritation from pancreaticobiliary reflux. This process drives sequential epithelial changes—from hyperplasia through dysplasia to carcinoma—often accompanied by molecular alterations such as K-ras and p53 mutations. Thus, this case illustrates the classic multistep carcinogenic cascade of chronic inflammation → metaplasia → dysplasia → carcinoma within the specific context of PBM. Through whole-exome sequencing of 15 GB-NEC cases, Liu et al. (51) revealed the first systematic characterization of their molecular profile. They found that GB-NECs are genetically distinct from gallbladder adenocarcinoma but resemble pulmonary LCNEC, featuring frequent mutations in TP53 (73%) and RB1 (27%). Importantly, the mutational landscape of GB-NEC revealed enrichment in Notch, WNT, and Hippo pathways, with clinically actionable alterations (e.g., ALK, BRCA1/2) identified in >85% of cases, supporting the rationale for targeted therapeutic strategies.

According to existing literature report (10–48), GB-LCNEC presents with non-specific symptoms like abdominal pain (73%), often coexisting with gallstones (47%) or chronic cholecystitis (22%), supporting its potential origin from inflammatory metaplasia (33, 37). It predominantly affects women aged 55–67 years. GB-LCNEC can occur in any anatomical part of the gallbladder (fundus, body, or neck), and the tumor diameter varies significantly (1–11.5 cm). Notably, larger tumors (diameter ≥ 2.5 cm) often present with diffuse gallbladder wall thickening (accounting for approximately 49%). The tumor shows high aggressiveness, with 29% exhibiting direct liver invasion and 51% developing liver metastases. An important histological feature of GB-LCNEC is that it is often accompanied by other epithelial malignant tumor components (accounting for 38% of reported cases), with adenocarcinoma being the most common (13 cases, 29%). Kaplan–Meier survival analysis revealed no significant difference in overall survival between the pure GB-LCNEC group and the impure GB-LCNEC group (i.e., GB-LCNEC accompanied by other epithelial malignant tumor components) (log-rank test, *P* = 0.55; analysis performed on our self-gathered online-reported case cohort (10–48). This indicates that the presence of other carcinoma components does not significantly affect the patients' survival (Supplementary Figure S2). Based on hormone-related symptoms, NENs are categorized as functional or non-functional (4). Fewer than 1% of GB-NEN cases present with carcinoid syndrome (52), such as diarrhea, facial flushing, hypoglycemia, and intractable peptic ulcers, which facilitate preoperative diagnosis (53). While only one case of ACTH-secreting GB-LCNEC has been reported (17), all other cases (including the two presented here) are non-functional NENs with atypical symptoms, which may reflect the small number of documented cases.

Tumor markers (e.g., CA19-9) and imaging examinations (ultrasound, CT, MRI) often lack specificity (54). CA19-9 was evaluable in only 10 cases (22%), and all showed elevated levels (Table 2). However, biliary obstruction or inflammation can also cause false positivity, making it unable to distinguish between benign and malignant conditions. Although published case reports have reported markedly elevated serum chromogranin-A (CgA) in patients with GB-LCNEC (35), existing clinical evidence is insufficient to validate it as a reliable and routine serum tumor marker. In our two cases, tissue CgA immunostaining was positive, confirming neuroendocrine differentiation; however, its role as a circulating marker remains uncertain and requires further investigation. Given these limitations, contrast-enhanced imaging serves as our primary preoperative examination. Scholars such as Jun et al. (11) have pointed out that there is no significant difference in CT findings between GB-SCNEC and GB-LCNEC. Nevertheless, contrast-enhanced CT/MRI can clearly demonstrate the depth of tumor invasion, involvement of adjacent structures, and intrahepatic or lymph node metastasis, providing a reliable basis for staging and surgical decision-making. Kim et al. (55) noted that the characteristic findings of GB-LCNEC include: well-defined borders, intact mucosa, and linear enhancement (a feature which may be related to the tumor originating from the submucosa). In clinical practice, if a gallbladder mass with well-defined borders and intact mucosa is detected, accompanied by liver or lymph node metastasis, the possibility of neuroendocrine carcinoma should be considered. Apart from these hallmarks, the tumor-enhancement pattern also exhibits obvious heterogeneity. Existing studies demonstrate that NENs typically show arterial-phase hyperenhancement (56). However, in our study, Case 1 displayed gradual heterogeneous enhancement with stronger uptake during the portal-venous phase, while the soft-tissue lesion at the gallbladder fundus in Case 2 presented obvious arterial-phase hyperenhancement. These observations suggest that GB-LCNEC has variable contrast-enhancement patterns on contrast-enhanced CT. These features may have a certain degree of specificity, but their value still needs to be confirmed by more studies.

Surgery is the first-line treatment option for GB-NENs (57), ranging from simple cholecystectomy to extensive radical resection consisting of hepatic resection and regional lymph-node dissection. A multicenter study by Tidjane et al. (58) demonstrated that standardized R0 radical resection with segment IVb/V hepatectomy and adequate lymphadenectomy yields comparable 5-year overall and disease-free survival rates between patients with advanced GB-NEC and those with gallbladder adenocarcinoma. Complete negative margins are critical for eradicating occult lesions, and postoperative adjuvant platinum-etoposide chemotherapy further prolongs median survival, highlighting the value of combined radical surgery and systemic therapy. In the second case reported in our study, the surgical scope failed to meet the guideline-recommended radical criteria and was therefore defined as suboptimal surgical treatment. To lower the risks of postoperative tumor recurrence and distant metastasis, the patient received standardized adjuvant chemotherapy and remained disease-free throughout 15 months of surveillance, confirming the survival advantage of adjuvant systemic therapy. For unresectable tumors, the primary approach is systemic therapy, the recommended first-line chemotherapy regimen consists of cisplatin or carboplatin combined with etoposide (59). However, a study by Iype et al. (14) found that the large cell subtype has a worse prognosis than the small cell subtype, and chemotherapy is more effective for SCNEC. Shimono et al. (15) reported a case of GB-LCNEC where the patient achieved 69-month survival through multimodal therapy, including preoperative arterial chemotherapy combined with three-dimensional radiotherapy, right hepatic trisegmentectomy, postoperative systemic chemotherapy, and gamma knife therapy for brain metastases. This case confirms the value of radiotherapy for local control in neoadjuvant/adjuvant settings. However, as shown in Table 2, except for this case (15), all other GB-LCNEC patients had a poor prognosis (median survival of 10 months), although a small number of patients achieved long-term survival (>3 years). This is presumably related to the timing of disease detection and the choice of treatment modality. Given the low preoperative diagnostic rate of GB-LCNEC and the close correlation between the choice of surgical approach and the pathological type, we recommend that intraoperative frozen section analysis be routinely performed—not only for cases highly suspicious for gallbladder carcinoma but also for patients with a preoperative diagnosis of gallbladder polyps or wall thickening—to guide subsequent therapeutic decisions. Meanwhile, medical institutions are advised to follow TNCD guidelines for postoperative follow-up, including physical examinations, tumor marker testing, and contrast-enhanced CT-based imaging. Follow-up visits are scheduled every 6 months within the first two postoperative years and annually thereafter. Tumor recurrence is confirmed by newly detected lesions on imaging or clinical manifestations consistent with tumor relapse (58).

## Conclusions

GB-LCNEC is highly aggressive and is frequently diagnosed at an advanced stage. The prognosis is poor, with a median survival of approximately 10 months. The median survival of the pure GB-LCNEC group was 26 months, while the median survival of the impure GB-LCNEC group cannot be reliably estimated due to limited sample size and follow-up time. Among the 43 GB-LCNEC cases documented in the literature, 14 deaths resulted from tumor progression. Both of our patients had pure GB-LCNEC and have survived longer than 10 months, with the maximum survival duration exceeding 19 months. Based on current evidence, we recommend: (1) strengthening early-stage diagnosis via multimodal imaging and avoiding preoperative biopsy for resectable gallbladder lesions to prevent tumor seeding; (2) prioritizing surgical feasibility assessment to achieve R0 resection; (3) initiating multicenter studies to explore effective adjuvant regimens; and (4) implementing close follow-up in high-risk patients to monitor for recurrence. This article, which presents two case reports and a literature review, enhances the understanding of this disease. In clinical practice, clinicians should maintain a high index of suspicion for suspected cases and perform a comprehensive preoperative evaluation to improve patient outcomes, while also paying special attention to patients with pancreaticobiliary maljunction. Future studies should focus on elucidating the molecular mechanisms of GB-LCNEC to inform the development of novel therapeutic strategies.

## Data Availability

The original contributions presented in the study are included in the article/Supplementary Material, further inquiries can be directed to the corresponding author.
