# Supplementary material for: Two cases of large cell neuroendocrine carcinoma of the gallbladder: case reports and systemic literature review
Source: Front Med (Lausanne). 2026 Sep 28;13:1885339. doi: 10.3389/fmed.2026.1885339 (PMC13654770; doi:10.3389/fmed.2026.1885339)
Supplement: Supplementary file 1 [file Datasheet1.docx]

**Supplementary Figure 1: Imaging Findings for Two Cases**

**Panels ①-③ correspond to Case 1**: **①** CT scan reveals a slightly hyperdense lesion with progressive enhancement; **②** MRI shows a hypointense lesion with marginal enhancement and restricted diffusion; **③** MRCP demonstrates a clump-like hypointense signal projecting into the gallbladder lumen.

**Panels ④-⑥ correspond to Case 2**: **④** CT scan shows an irregular soft tissue shadow with obvious enhancement; **⑤** MRI shows isointense signal on T1-weighted imaging, nodular filling defect on T2-weighted imaging, and slightly hyperintense signal on DWI; **⑥** Segmental focal dilatation along the course of the common bile‑duct. ***Note****: The red arrows indicate the tumor masses; and the blue arrow indicates common bile‑duct dilatation.*

**Supplementary Figure 2: Comparison of overall survival between patients with pure and impure GB-LCNEC**

**Supplementary Table 1: Differential Diagnosis of GB-LCNEC**

| **Key Differentiation Points** | **GB-LCNEC** | **CB-SCNEC** | **GB-MiNEN** | **Poorly Cohesive Adenocarcinoma** |
| --- | --- | --- | --- | --- |
| **Tissue Structure** | Organoid/nested/rosette/palisading/trabecular arrangement, separated by fibrovascular stroma | Same as LCNEC | Bicompartmental structure: neuroendocrine component (NET/NEC-like) + non-neuroendocrine component (e.g., glandular ducts, keratin pearls); Demarcation: clear or mutually infiltrative | Diffuse/sheet-like or nested growth, lacking neuroendocrine organoid architecture |
| **Cellular Morphology** | Large cells (diameter > 3 lymphocytes), abundant cytoplasm; coarse granular chromatin, prominent nucleoli (most reliable) | Small cells (1-2 lymphocytes in diameter), Scant cytoplasm ("naked nucleus" appearance), Finely granular ("salt-and-pepper") chromatin, Inconspicuous nucleoli, Frequent nuclear molding | Neuroendocrine component: NET/NEC-like; Non-neuroendocrine component: e.g., adenocarcinoma/squamous cell carcinoma; Nuclear features: Reflect respective tumor origins | Intermediate to large cells; Marked pleomorphism; Prominent nuclear atypia; Discernible nucleoli |
| **Ki67 Index & Proliferative Activity** | Ki-67 >20%; Mitotic rate >20/2mm² | Same as LCNEC | Two components evaluated separately; neuroendocrine component usually with high Ki-67 | variable Ki-67 index; Moderate proliferative activity |
| **IHC** | Neuroendocrine markers (CgA, Syn, CD56, etc.) diffusely positive; CKpan/Cam5.2: paranuclear dot-like positive; MUC5AC/CDX2 usually negative | Neuroendocrine markers consistent with LCNEC; epithelial markers may show focal weak positivity; TTF-1 for auxiliary diagnosis | Neuroendocrine component: Syn, CgA, etc., positive; Non-neuroendocrine component: CK7, CK20, CDX2, etc., positive | Neuroendocrine markers negative or only scattered positive; CKpan/Cam5.2: diffuse strong positivity; MUC5AC/CDX2 often positive |
| **Key Points** | Large cells; prominent nucleoli; mitotic rate >20/2mm²; Ki-67 >20%; need to rule out MiNEN and distinguish from SCNEC | Small cells; inconspicuous nucleoli; mitotic rate >20/2mm²; Ki-67 >20%; need to distinguish from LCNEC | Bicompartmental (each component >30%), with immunohistochemical features corresponding to their respective components | Lack of neuroendocrine differentiation (Syn negative); diffuse CK positivity |
| **Diagnostic Considerations** | May be combined with adenocarcinoma or squamous cell carcinoma (if combined component <30%, still classified as LCNEC); combined adenocarcinoma component easily misdiagnosed as MiNEN or poorly cohesive adenocarcinoma; multiple sampling required to confirm single component | If large cell proportion ≤10%, classified as SCNEC; crush artifact mimics lymphoma; TTF-1 positivity easily mistaken for pulmonary origin | Must contain non-neuroendocrine component (e.g., adenocarcinoma/squamous cell carcinoma), with each component >30%; biopsy specimens may only sample a single component, in which case report: "contains at least ×× component; recommend excluding MiNEN after surgery" | When morphology overlaps with LCNEC, CK positivity and Syn negativity are sufficient to rule out (LCNEC) |
